# Transient receptor potential canonical 5 (TRPC5) protects against pain and vascular inflammation in arthritis and joint inflammation

**DOI:** 10.1136/annrheumdis-2015-208886

**Published:** 2016-05-10

**Authors:** Khadija M Alawi, Fiona A Russell, Aisah A Aubdool, Salil Srivastava, Yanira Riffo-Vasquez, Lineu Baldissera, Pratish Thakore, Nurjahan Saleque, Elizabeth S Fernandes, David A Walsh, Susan D Brain

**Affiliations:** 1 Cardiovascular Division, BHF Cardiovascular Centre of Excellence and Centre of Integrative Biomedicine, King's College London, London, UK; 2 Sackler Institute of Pulmonary Pharmacology, Institute of Pharmaceutical Science, King's College London, London, UK; 3 Institute of Pharmaceutical Science, King's College London, London, UK; 4 Programa de Pós-Graduação, Universidade Ceuma, São Luís, Brazil; 5 Department of Academic Rheumatology, Arthritis Research UK Pain Centre, University of Nottingham, City Hospital, Nottingham, UK

**Keywords:** Synovitis, Arthritis, Inflammation

## Abstract

**Objective:**

Transient receptor potential canonical 5 (TRPC5) is functionally expressed on a range of cells including fibroblast-like synoviocytes, which play an important role in arthritis. A role for TRPC5 in inflammation has not been previously shown in vivo. We investigated the contribution of TRPC5 in arthritis.

**Methods:**

Male wild-type and TRPC5 knockout (KO) mice were used in a complete Freund's adjuvant (CFA)-induced unilateral arthritis model, assessed over 14 days. Arthritis was determined by measurement of knee joint diameter, hindlimb weightbearing asymmetry and pain behaviour. Separate studies involved chronic pharmacological antagonism of TRPC5 channels. Synovium from human postmortem control and inflammatory arthritis samples were investigated for TRPC5 gene expression.

**Results:**

At baseline, no differences were observed. CFA-induced arthritis resulted in increased synovitis in TRPC5 KO mice assessed by histology. Additionally, TRPC5 KO mice demonstrated reduced ispilateral weightbearing and nociceptive thresholds (thermal and mechanical) following CFA-induced arthritis. This was associated with increased mRNA expression of inflammatory mediators in the ipsilateral synovium and increased concentration of cytokines in synovial lavage fluid. Chronic treatment with ML204, a TRPC5 antagonist, augmented weightbearing asymmetry, secondary hyperalgesia and cytokine concentrations in the synovial lavage fluid. Synovia from human inflammatory arthritis demonstrated a reduction in TRPC5 mRNA expression.

**Conclusions:**

Genetic deletion or pharmacological blockade of TRPC5 results in an enhancement in joint inflammation and hyperalgesia. Our results suggest that activation of TRPC5 may be associated with an endogenous anti-inflammatory/analgesic pathway in inflammatory joint conditions.

## Introduction

Rheumatoid arthritis (RA) is a chronic, systemic autoimmune disease characterised by inflammation of diarthrodial joints, joint tenderness and swelling.^1^ RA affects 1% of the worldwide population and initiates with inflammation of the synovium in peripheral joints, which progresses to destruction of articular cartilage, leading to significant joint degeneration, pain and loss of function.^1^ ^2^ Transient receptor potential (TRP) channels are non-selective cation channels, which are involved in divergent somatosensory functions, including pain.^3^ Several members of TRP channels, in particular, TRP vanilloid 1 and TRP ankyrin 1 , play a detrimental role in inflammatory pain conditions, including arthritis,^4^ in addition to being involved in vascular regulation.^5^ We have shown that pharmacological blockade or genetic deletion of these channels is associated with an improved outcome of adjuvant-induced arthritis in mice.^4^

TRP canonical 5 (TRPC5) is a member of the canonical family of TRP channels that assemble to form non-selective cation channels as homo-tetramers or hetero-tetramers.^3^ TRPC5 more commonly associates with other members of TRPC channels, notably, TRPC1 and TRPC4.^6^ TRPC5 is expressed in the central nervous system^7^ and peripherally in sensory nerves.^8^ ^9^ While there is limited evidence of a functional role in arthritis in vivo, TRPC5, together with TRPC1, are expressed in CD55^+^ fibroblast-like synoviocytes (FLS).^10^ Stimulation of TRPC5 by the endogenous agonist, thioredoxin, results in a suppression of matrix metalloproteinases (MMPs) secretion in both humans and animal FLS, highlighting a conserved effect. Furthermore, blockade of TRPC5 by antibody or siRNA treatment potentiated MMP-2 secretion from FLS of patients with RA.^10^

Pharmacological tools for TRPC5 channels are limited;^6^ however, a TRPC4/5 antagonist, ML204, was characterised as selective antagonist with 19-fold selectivity over TRPC6.^11^ ML204 exhibited stability in vitro, with a half-life of 2 h and was also functionally effective in vivo.^12^ Global TRPC5 knockout (KO) mice show normal survival, fertility and growth compared with wild-type (WT) control mice;^7^ however, the functional significance of TRPC5 in inflammatory joint disease is unclear. We hypothesised that global deletion or pharmacological antagonism of TRPC5 would exacerbate joint disease associated with increased inflammation and pain.

## Materials and methods

The full methods are provided in the online supplementary material.

10.1136/annrheumdis-2015-208886.supp1 Supplementary data

### Mice

Male, age-matched 129S1/SvImJ TRPC5 WT and TRPC5 KO bred from established breeding pairs^7^ were used at 8–12 weeks of age. All experiments were conducted under United Kingdom Home Office Animals (Scientific Procedures) Act 1986 and approved by the King's College London Animal Care and Ethics Committees.

### Induction of arthritis

The complete Freund's adjuvant (CFA)-induced unilateral arthritis model was investigated over 14 days, as characterised previously.^4^ Behavioural measurements of hyperalgesia were obtained at baseline and stated timepoints.

### Histology and immunofluorescence staining

Histological and immunofluorescence staining was performed as previously described.^4^ ^13^

### Human tissue sample collection

All live and postmortem (PM) donations were obtained at Sherwood Forest Hospitals NHS Foundation Trust, Sutton-in-Ashfield, UK. Synovium from the knee was collected during arthroplastic surgery or at the time of PM examination and stored at −80°C until RNA extraction. Three groups, non-arthritic cases, RA and osteoarthritic (OA) cases, were selected to be age and, if possible, sex matched, as previously described.^14^ Patient details including inflammatory scores are described in online supplementary table S1.

### Statistical analysis

Results are expressed as mean±SEM and analysed by two-way analysis of variance and Bonferroni post hoc test using Graph Pad Prism V.5.0 (San Diego, California, USA) unless stated. For non-parametric data (human mRNA expression), results were analysed using Kruskal–Wallis test followed by post hoc Dunn's comparison. Significance was accepted as p<0.05.

## Results

### Deletion of TRPC5 exacerbates chronic CFA-induced arthritis

**Figure 1:**
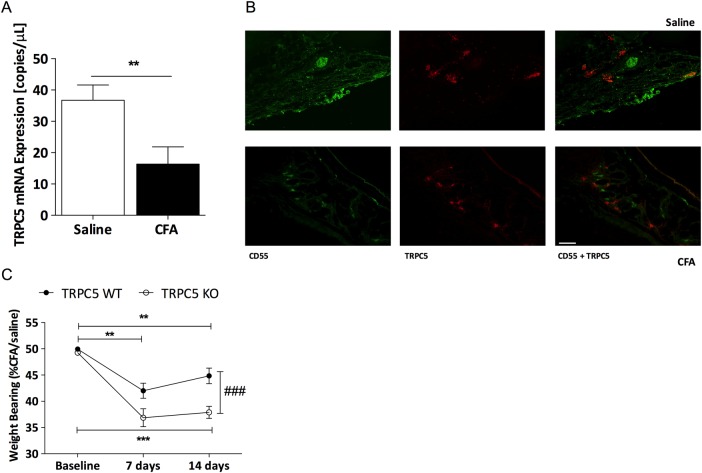
Transient receptor potential canonical 5 (TRPC5) mRNA expression is regulated in the arthritic synovium; evidence that deletion exacerbates complete Freund's adjuvant (CFA)-induced arthritis. (A) Real-time PCR analysis of TRPC5 expression in the synovial membrane of wild-type (WT) mice 14 days following saline or CFA, normalised to hypoxanthine-guanine phosphoribosyltransferase (HPRT), β-actin and PLA_2_; n=7. (B) Representative double immunofluorescence staining of CD55 (green) and TRPC5 (red) in normal and arthritic synovium, scale bars represent 50 µm. (C) Time-course analysis of weightbearing asymmetry following induction of arthritis in TRPC5 WT (n=9) and TRPC5 knockout (KO) (n=8) mice. *p<0.05, **p<0.01, ***p<0.001 vs control; ###p<0.001 vs WT by two-tailed Student's test (A) and two-way analysis of variance+Bonferroni post hoc test (C); values are mean±SEM.

We examined the mRNA expression of TRPC5 in the mouse synovium 14 days following CFA-induced arthritis and observed a significant reduction in the expression of TRPC5 in the ipsilateral synovium compared with the contralateral synovium in WT mice (p<0.01; figure 1A). This reduction in TRPC5 expression was not associated with regulation of associated members of TRPC channels as no significant difference was observed in the expression of TRPC1, TRPC3 or TRPC6 (see online supplementary table S4) and we did not detect TRPC4 expression. Double-immunofluorescence labelling illustrated the expression of TRPC5 in the intimal and subintimal lining of the mouse synovium (figure 1B), with co-localisation with CD55 observed mostly in the intimal lining, as previously described.^10^ The functional contribution of TRPC5 in vivo was investigated by assessing mobility and movement-related pain over a period of 14 days, following CFA-induced arthritis. We found a significant reduction in the ipsilateral hindlimb weightbearing of WT mice (approximately 10%) compared with baseline by day 7, which was modestly ameliorated by day 14 (figure 1C). Genetic deletion of TRPC5 resulted in an enhanced discomfort and exacerbated weightbearing asymmetry on day 14 (approximately 10%), compared with WT mice.

### TRPC5 regulates hyperalgesia in CFA-induced arthritis

**Figure 2:**
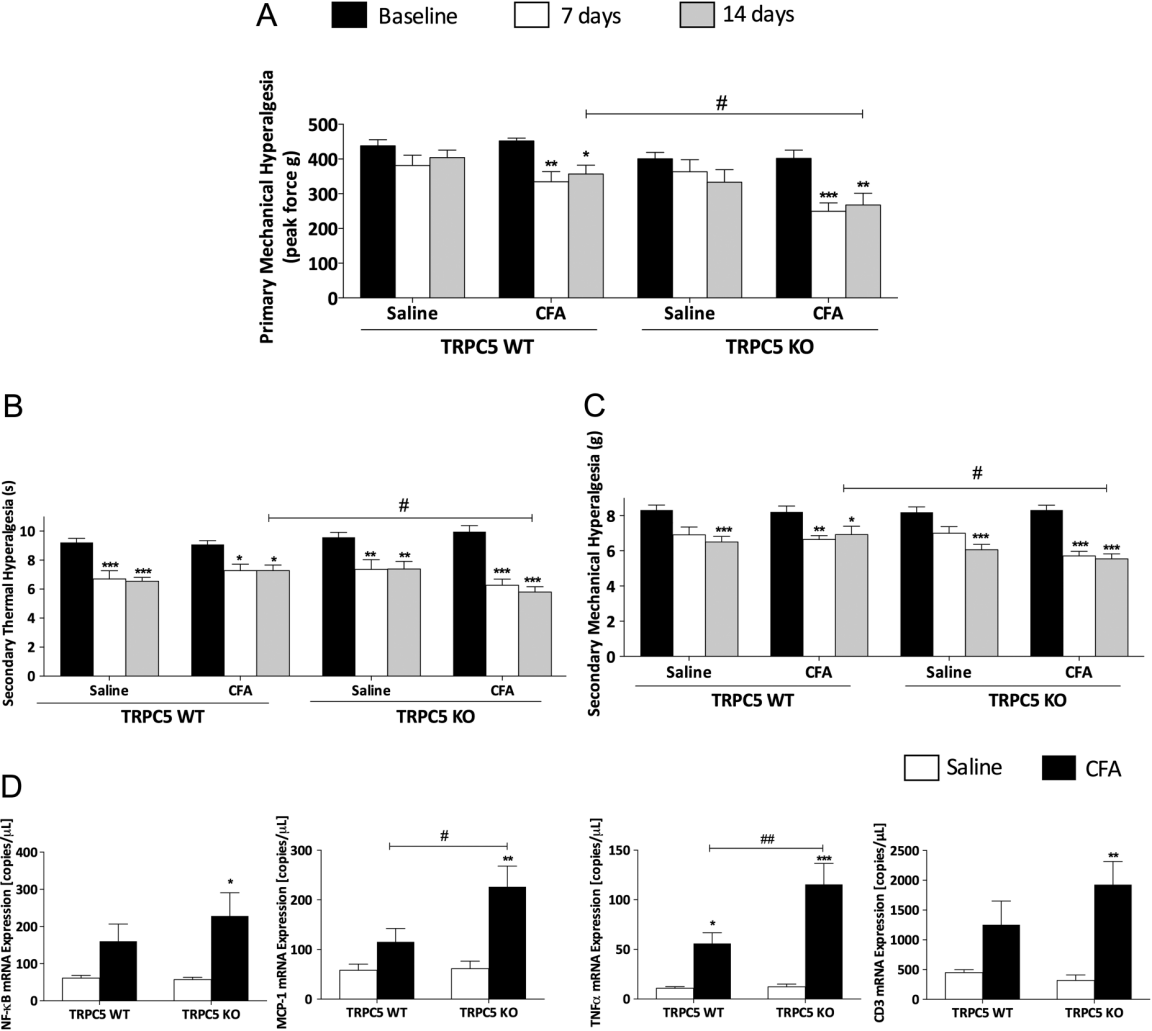
Nociceptive parameters assessment in complete Freund's adjuvant (CFA)-induced arthritis in wild-type (WT) and transient receptor potential canonical 5 (TRPC5) knockout (KO) mice. (A) Time-course analysis of primary hyperalgesia of the hindlimb in TRPC5 WT (n=9) and KO (n=8) mice. (B) Secondary thermal hyperalgesia assessed before and weekly following CFA-induced arthritis in TRPC5 WT and KO mice. (C) Secondary mechanical hyperalgesia in the hindpaw assessed before and weekly following CFA-induced arthritis in TRPC5 WT and KO mice. (D) Real-time quantitative PCR analysis of the expression of inflammatory mediators: nuclear factor of kappa light polypeptide gene enhancer in B cells (NF-κB), monocyte chemoattractant protein-1 (MCP-1), tumour necrosis factor α (TNFα), cluster of differentiation 3 (CD3) in the contralateral and ipsilateral synovium of TRPC5 WT (n=8) and KO (n=7) mice, normalised to hypoxanthine-guanine phosphoribosyltransferase (HPRT), β-actin and PLA_2_. *p<0.05, **p<0.01, ***p<0.001 vs control; #p<0.05, ##p<0.01 vs WT by two-way analysis of variance +Bonferroni post hoc test; values are mean±SEM.

Hyperalgesia encompassing primary hyperalgesia of the affected joint and secondary hyperalgesia of distal, unaffected sites are a common symptom in RA and in rodent models of arthritis.^4^ ^15^ We measured primary hyperalgesia of the hindknee joint, and secondary hyperalgesia in the hindpaw of WT and TRPC5 KO mice following CFA-induced arthritis. At baseline, no difference was observed between the groups (figure 2A–C). Over a period of 14 days, WT and TRPC5 KO mice developed primary hyperalgesia of the knee joint (figure 2A) and this was exacerbated in TRPC5 KO compared with WT mice (p<0.05). Following CFA-induced arthritis, bilateral thermal hyperalgesia was observed in both groups with augmented ipsilateral hyperalgesia in TRPC5 KO mice (figure 2B, p<0.05). Similarly, secondary mechanical allodynia of the ipsilateral hindpaw (figure 2C) was enhanced in TRPC5 KO compared with WT mice (p<0.05).

We investigated the mRNA expression of inflammatory mediators in the synovium 14 days following CFA-induced arthritis and observed a significant induction in the expression of the nuclear transcription factor kappa B in the ipsilateral synovium of TRPC5 KO mice (figure 2D). Similarly, we observed a potentiated induction in the expression of inflammatory mediators such as monocyte chemotactic protein 1 and tumour necrosis factor α (TNFα) in TRPC5 KO mice compared with WT mice, suggesting augmented synovitis. However, the expression of the T cell marker, cluster of differentiation 3, was significantly increased in both TRPC5 WT and KO mice highlighting increased infiltration of lymphocytes following CFA-induced arthritis.

### Deletion of TRPC5 enhances synovitis and local inflammation

**Figure 3:**
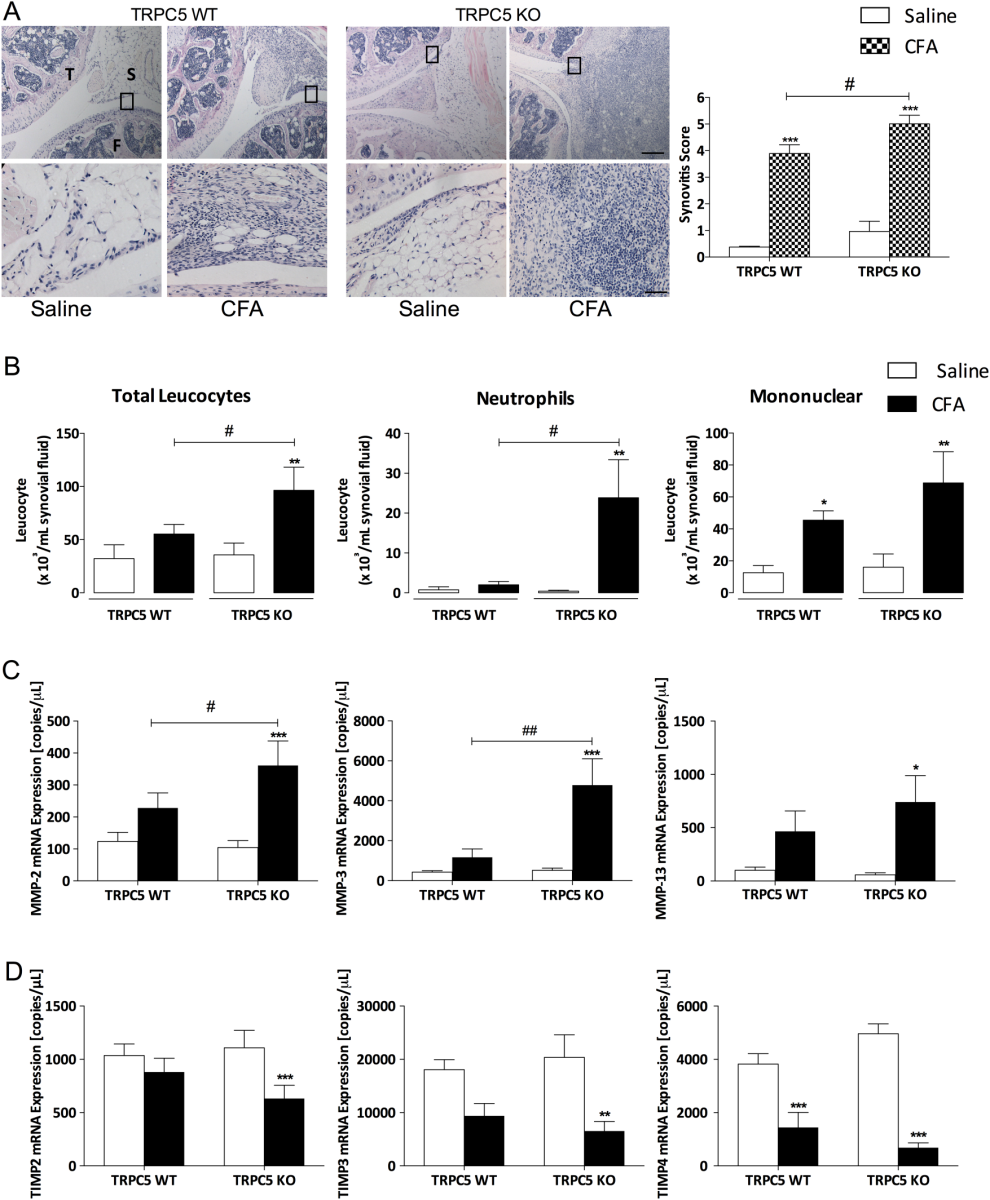
Genetic deletion of transient receptor potential canonical 5 (TRPC5) enhances synovitis and cellular infiltration following complete Freund's adjuvant (CFA)-induced arthritis. (A) Histological assessment of synovitis assessed by H&E staining; top panel shows the tibiofemoral junction (×4) 14 days following saline or CFA injection, inset shows higher power magnification (×20) below. Each knee shown is a representative for a group of mice (n=5–6); scale bars (×4) represents 200 µm, (×20) represents 50 µm; T, tibia; F, femur; S, synovium. (B) Cytospin preparations of synovial lavage fluid of wild-type (WT) and TRPC5 knockout (KO) mice illustrating cellular infiltration 14 days following CFA-induced arthritis and leucocyte population analysis. (C) Real-time quantitative PCR analysis of the expression of matrix metalloproteinases (MMP-2, MMP-3, MMP-13) in the ipsilateral synovium compared with the contralateral synovium in TRPC5 WT (n=8) and KO (n=7) mice. (D) Expression of tissue inhibitors of MMP (TIMP-2, TIMP-3, TIMP-4) in the ipsilateral synovium compared with the contralateral synovium in TRPC5 WT (n=8) and KO (n=7) mice, normalised to hypoxanthine-guanine phosphoribosyltransferase (HPRT), β-actin and PLA2..*p<0.05, **p<0.01, ***p<0.001 vs control; #p<0.05, ##p<0.01 vs WT by two-way analysis of variance+Bonferroni post hoc test; values are mean±SEM.

Synovitis, characterised by synovial inflammation, is a hallmark of arthritic joint conditions and is predominantly driven by resident FLS, which contribute to the inflammation by recruiting and activating immune cells.^16^ We investigated joint histopathology 14 days following CFA-induced arthritis and observed significant synovitis in WT mice, as illustrated by thickening of the synovium lining, cellularisation and infiltration (figure 3A). Deletion of TRPC5 resulted in a significantly increased synovitis score compared with WT CFA (p<0.05). Assessment of the leucocyte population of synovial lavage fluid by cytospin preparations illustrated an increase in mononuclear cell infiltrate in the synovial fluid of WT mice 14 days following CFA-induced arthritis (figure 3B). By contrast, TRPC5 KO mice exhibited a predominant infiltration of neutrophils (figure 3B) while circulating leucocytes demonstrated no significant difference compared with WT mice (see online supplementary figure S2).

We investigated the mRNA expression of MMPs with an established role in arthritic conditions,^17^ ^18^ such as MMP-2, MMP-3 and MMP-13. Results demonstrated a significant induction in the expression of these enzymes in the ipsilateral synovium of TRPC5 KO mice (figure 3C). Additionally, the expression of the endogenous inhibitors of MMPs and tissue inhibitors of metalloproteinases was investigated, which demonstrated a significant reduction in the expression of tissue inhibitors of MMP (TIMP)-2 and TIMP-3 in the ipsilateral synovium of TRPC5 KO mice (figure 3D; p<0.01 and <0.001, respectively), while the expression of TIMP-4 was reduced in the ipsilateral synovium of both WT and TRPC5 KO mice (p<0.001).

### Chronic pharmacological antagonism of TRPC5 exacerbates inflammation

**Figure 4:**
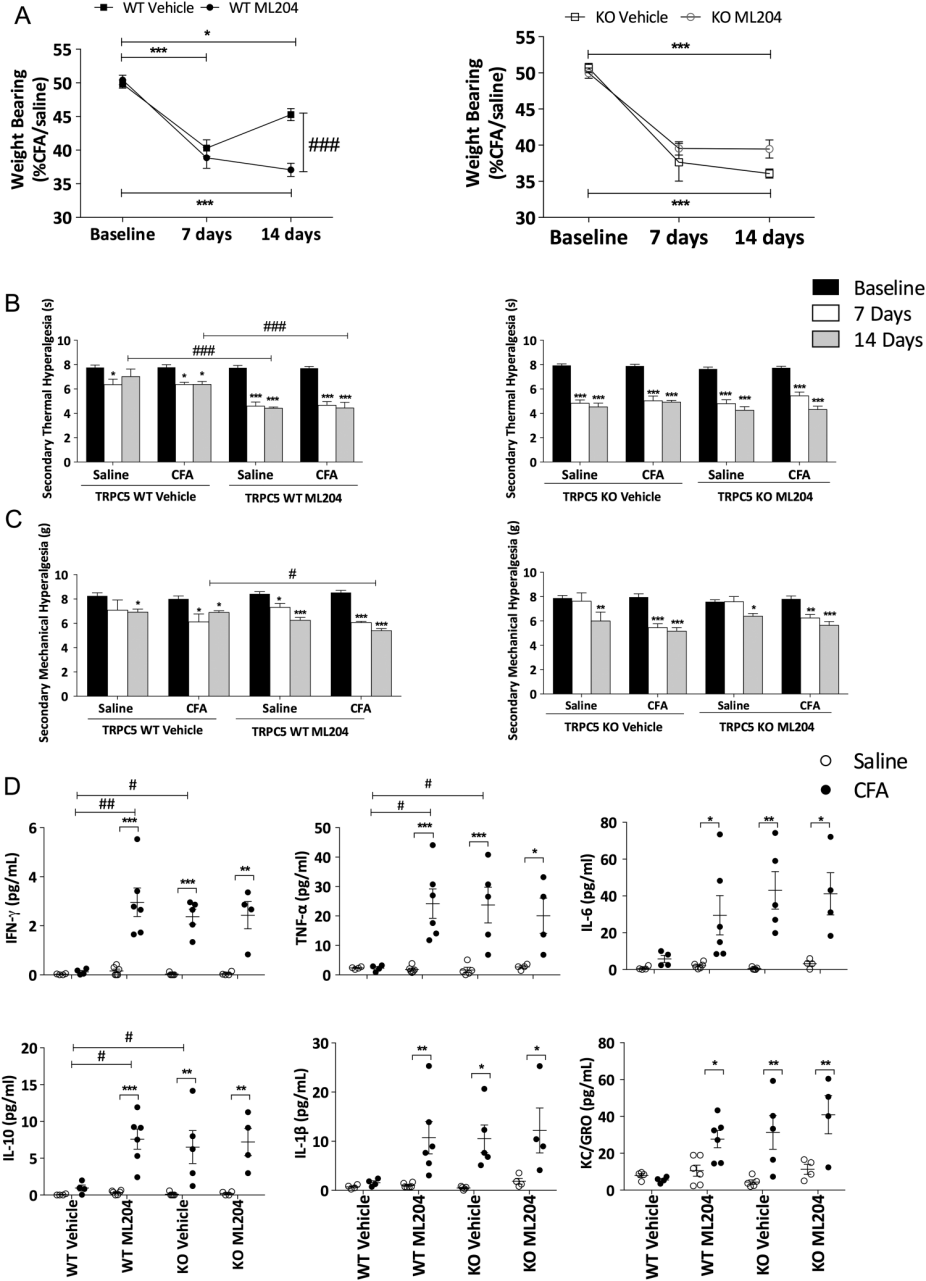
Chronic pharmacological blockade of transient receptor potential canonical 5 (TRPC5) exacerbates complete Freund's adjuvant (CFA)-induced arthritis in wild-type (WT) mice. (A) Time-course analysis of weightbearing asymmetry in vehicle (2% dimethyl sulfoxide (DMSO) in saline, intraperitoneally)-treated WT and TRPC5 knockout (KO) mice (n=5), ML204-treated WT and TRPC5 KO mice (2 mg/kg, intraperitoneally, daily n=6). (B) Secondary thermal hyperalgesia assessed before and weekly following CFA-induced arthritis in TRPC5 WT and KO mice treated with vehicle or ML204. (C) Secondary mechanical hyperalgesia in the hindpaw assessed before and weekly following CFA-induced arthritis in TRPC5 WT and KO mice treated with vehicle or ML204. (D) Cytokine concentrations in the synovial lavage fluid 14 days following CFA-induced arthritis in TRPC5 WT and KO mice treated with vehicle or ML204; interferon-γ (IFN-γ), tumour necrosis factor-α (TNFα), interleukin (IL)-6, IL-10 and IL-1β, keratinocyte chemoattractant (KC). *p<0.05, **p<0.01, ***p<0.001 vs baseline (A–C) and versus control (E); #p<0.05, ##p<0.01, ###p<0.001 vs WT vehicle by two-way analysis of variance+Bonferroni post hoc test; values are mean±SEM.

To confirm the findings obtained in genetically modified mice, we used the TRPC4/5 antagonist, ML204.^11^ ML204 or vehicle was administered 1 h prior to induction of arthritis and daily thereafter (see online supplementary figure S3A). Chronic treatment with ML204 had no effect on body weight (see online supplementary figure S3B), but significantly augmented joint inflammation, hyperalgesia and weightbearing asymmetry in WT mice (figure 4A–C). We investigated the selectivity of ML204 by performing parallel experiments in TRPC5 KO mice and noted no differences between TRPC5 KO mice treated with ML204 or vehicle (figure 4A–C). Cytokine concentrations (interferon (IFN)-γ, TNFα and interleukin (IL)-10) in the ipsilateral synovial fluid of WT mice treated with ML204 were increased compared with ipsilateral synovial fluid of vehicle-treated WT mice (figure 4D). IL-6, IL-1β and the chemokine, keratinocyte chemoattractant, exhibited a similar trend (not significant). Moreover, TRPC5 KO mice treated with vehicle or ML204 showed no difference in cytokine concentrations; however, IFN-γ, TNFα and IL-10 concentrations were augmented in TRPC5 KO mice compared with vehicle-treated WT mice. Circulating cytokines were also investigated (see online supplementary figure S4), and no distinct differences were observed in all groups.

### TRPC5 regulates synovial vascularity and joint oedema

**Figure 5:**
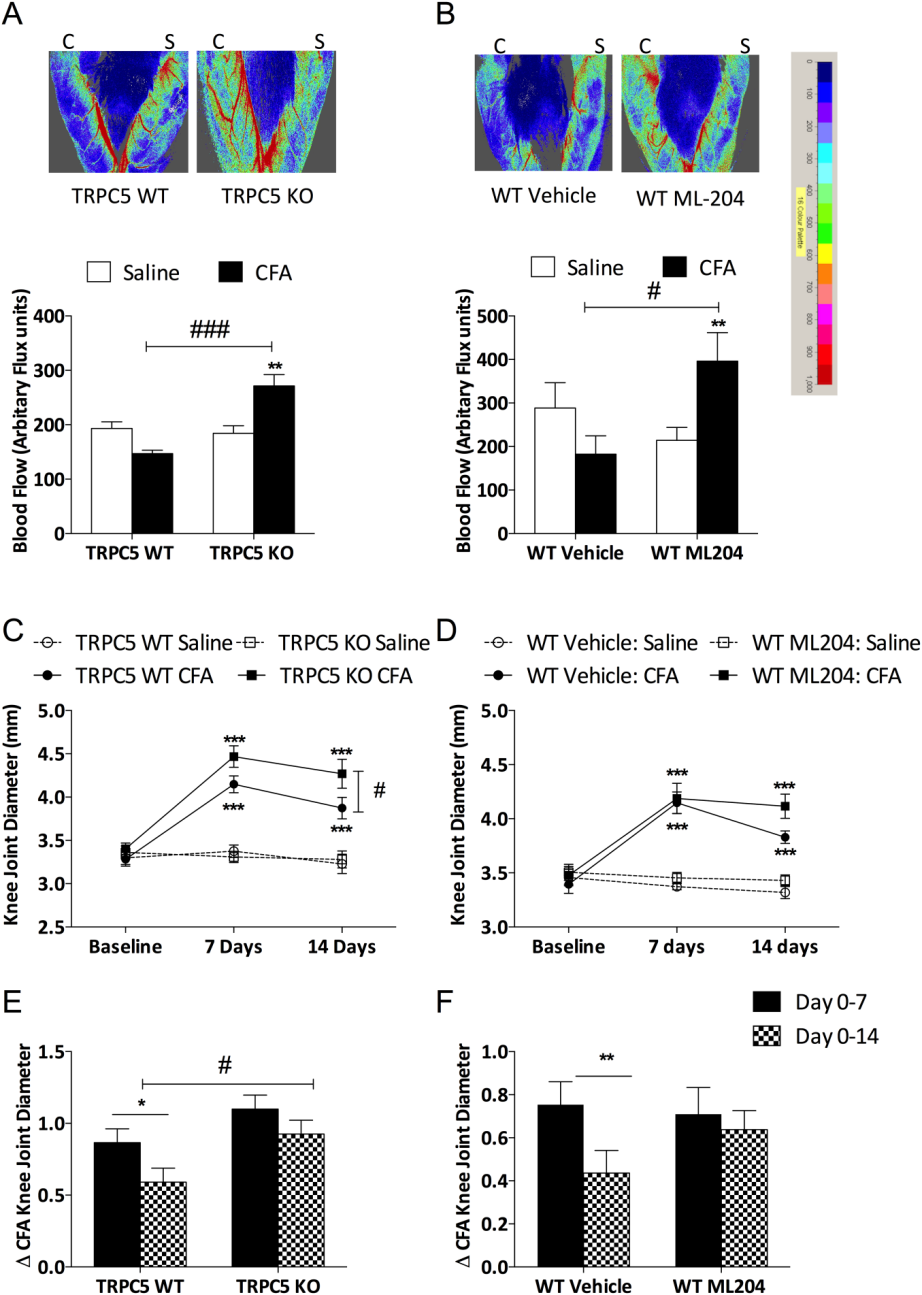
Augmented synovial vascularity and swelling in transient receptor potential canonical 5 (TRPC5) knockout (KO) and antagonist-treated wild-type (WT) mice. (A) Top panel: representative flux/blood flow image obtained by full-field laser perfusion imaging of blood flow in the synovial membrane day 14 following complete Freund's adjuvant (CFA)-induced arthritis in TRPC5 WT (n=7) and TRPC5 KO (n=7) mice with graphical representation of the results. (B) Synovial blood flow (top panel) assessed on day 14 following CFA-induced arthritis in vehicle (2% dimethyl sulfoxide (DMSO) in saline, intraperitoneally)-treated WT (n=5) and ML204-treated mice (2 mg/kg, intraperitoneally, daily n=6) on day 14 following CFA-induced arthritis with graphical representation of the results. (C) Time course of knee diameter swelling following CFA-induced arthritis in TRPC5 WT (n=10) and KO (n=10) mice and (E) delta (Δ) change in knee joint swelling. (D) Time course of joint swelling following CFA-induced arthritis in vehicle (2% DMSO in saline, intraperitoneally)-treated WT (n=5) and ML204-treated mice (2 mg/kg, intraperitoneally, daily n=6) following CFA-induced arthritis and (F) delta (Δ) change in knee joint diameter. *p<0.05, **p<0.01, ***p<0.001 vs control (A and B) versus baseline (C and D); ##p<0.01 vs WT by two-way analysis of variance+Bonferroni post hoc test; values are mean±SEM.

Enhanced synovial vascularity is an indicator of disease activity in RA^19^ ^20^ and has been determined in rodent models.^21^ ^22^ We investigated synovial blood flow following CFA-induced arthritis by laser speckle imaging. The results demonstrated that following CFA-induced arthritis, the ipsilateral synovial membrane was significantly vascularised on day 7 compared with the contralateral synovium. In contrast, synovial blood flow on day 14 showed a modest reduction in the ipsilateral synovium, although this did not reach significance (see online supplementary figure S5). At day 14 following CFA-induced arthritis in WT and TRPC5 KO mice, we observed no significant change in blood flow of the ipsilateral synovium of TRPC5 WT mice compared with the contralateral synovium. Conversely, blood flow was significantly increased in the ipsilateral synovium of TRPC5 KO mice compared with the contralateral (p<0.01) and with WT mice (p<0.001) (figure 5A). Consistent with previous results, chronic TRPC5 antagonism with ML204 closely mimicked the TRPC5 KO results, whereby ipsilateral synovial blood flow was significantly increased compared with the contralateral synovium and with vehicle-treated WT mice (figure 5B).

We assessed joint swelling by measuring thickness following CFA-induced arthritis and observed a significant increase by day 7 in both WT and TRPC5 KO mice; however, swelling was augmented in TRPC5 KO mice on day 14 compared with WT mice (figure 5C; p<0.05). Assessment of joint diameter compared with baseline at days 7 and 14 illustrated a significant amelioration (day 14 vs day 7, p<0.01) in TRPC5 WT mice; this was not observed in TRPC5 KO mice, where the mean change in knee joint diameter was significantly higher compared with WT mice (figure 5E; p<0.05). TRPC5 WT mice treated with ML204 did not show a significant difference compared with vehicle-treated WT mice (figure 5D); however, vehicle-treated WT mice displayed resolution of joint swelling by day 14 (p<0.01), and this was absent in ML204-treated WT mice (figure 5F).

### Regulation of TRPC5 expression in human arthritis

**Figure 6:**
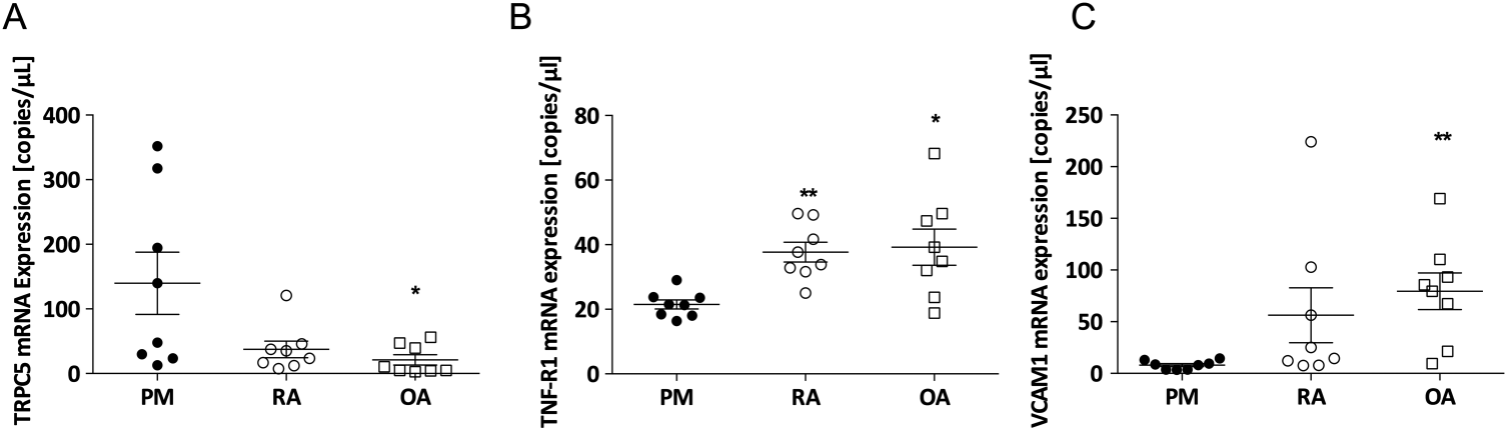
Expression of transient receptor potential canonical 5 (TRPC5) in human synovium under normal and inflammatory arthritis conditions. Real-time quantitative PCR analysis of the expression of (A) TRPC5, (B) tumour necrosis factor receptor 1 (TNF-R1) and (C) vascular cell adhesion molecule (VCAM-1) in the synovium of postmortem (PM) control, rheumatoid arthritis (RA) and osteoarthritis (OA) normalised to β-actin, B_2_M, and RPL13A. *p<0.05 vs control, as determined by Kruskal–Wallis test followed by Dunn's post hoc comparison; values are mean±SEM.

We investigated the mRNA expression of TRPC5 in human synovium under normal and inflammatory conditions encompassing RA and OA (see online supplementary table S1 for patient details and inflammation scores). We detected TRPC5 expression in control synovium (figure 6A), and similar to results observed in our findings in mice, TRPC5 expression exhibited a trend towards a reduction in RA samples; however, this was not significant. In contrast, TRPC5 expression was significantly reduced in OA samples (p<0.05) compared with non-arthritic controls. In contrast, the expression of the type 1 TNF receptor and vascular adhesion molecule-1 were significantly increased in arthritic synovium compared with the control synovium (p<0.05; figure 6B, C), paralleling increased inflammatory responses in these samples, and the histological synovitis scores (see online supplementary table S1).

## Discussion

In the present study, we investigated the in vivo contribution of TRPC5 to the local inflammatory changes that occur in arthritis using pharmacogenomic approaches. We show that genetic deletion or blockade of TRPC5 in arthritic mice resulted in marked exacerbation of hyperalgesia and, critically, increased localised inflammation in the synovium characterised by increased cellular infiltration, secretion of early response cytokines and enhanced synovial vascularisation. This provides the first in vivo evidence to show that TRPC5 acts as a regulatory channel to protect the joint against inflammatory insults.

Joint pain remains an unmet clinical need,^1^ and patients with RA exhibit symmetry in both their clinical symptoms and pain. Peripheral and central sensitisation, characterised by pain radiating to an unaffected site, is a common feature.^4^ ^15^ ^23^ We designed experiments to investigate this by inducing unilateral arthritis, which resulted in primary hyperalgesia, in addition to symmetrical secondary hyperalgesia. We confirmed the development of primary hyperalgesia through measurement of joint withdrawal threshold to pressure application and weightbearing asymmetry; both parameters were principally unilateral in nature and significantly exacerbated in TRPC5 KO mice.

We investigated central sensitisation^1^ by measuring nociceptive responses in the hindpaw and show the development of bilateral hyperalgesia following intra-articular injection of CFA in the hindknee joint. While the contralateral secondary hyperalgesia was not influenced by TRPC5 deletion/antagonism, secondary ipsilateral hyperalgesia was significantly enhanced in TRPC5 KO and WT antagonist-treated mice. The role of TNFα in inflammatory hyperalgesia is fundamental^23–25^; we detected abundant expression of TNFα mRNA in the inflamed synovium of both TRPC5 WT and KO mice, 14 days following CFA-induced arthritis. However, the induction of TNFα was substantially elevated in TRPC5 KO mice. Furthermore, assessment of TNFα concentrations in synovial lavage fluid was in agreement with the mRNA studies, highlighting enhanced synthesis and secretion of TNFα following deletion of TRPC5. As a potent pro-algesic mediator, this increase in TNFα availability may facilitate the augmented hyperalgesia observed in TRPC5 KO mice.

Synovitis, characterised by synovial hyperplasia and inflammation, is a hallmark of RA, with a prominent role for FLS.^14^ Here, we show positive TRPC5 expression in the mouse synovium and co-localisation with CD55. CD55-positive staining was observed in the intimal lining layer, while TRPC5 was found both in the intimal and subintimal lining. Co-localisation of TRPC5 with CD55 was previously shown in human RA synovium^10^; however, the transcriptional expression of TRPC5 under joint inflammation was previously unknown. Thus, we investigated the mRNA expression of TRPC5 and demonstrated a reduction in inflamed human arthritis samples. Interestingly, downregulation of TRPC5 was pronounced in synovium from patients with OA, highlighting a potential role for TRPC5 in OA. However, this remains to be investigated. Collectively, the results were in agreement with our mouse model of CFA-induced arthritis, revealing an association between TRPC5 expression and joint inflammation. Indeed, genetic deletion of TRPC5 significantly increased the synovitis score in mice, as illustrated by significantly increased cellularity of resident and infiltrating cells.

Hyperpermeability, angiogenesis and vascular remodelling contribute to the inflammatory response in arthritis.^20^ ^26^ ^27^ Altered synovial vascularisation is dependent on disease activity,^26^ ^28^ where increased vascularisation reflects an increase in sensory innervation and pain,^29^ ^30^ while in chronic RA, the synovium exhibits hypoperfusion.^31^ In line with this, we show that in WT mice, synovial vascularisation was pronounced on day 7, but unchanged compared with the contralateral synovium by day 14. A matched assessment of synovial blood flow in TRPC5 WT and KO mice revealed a different profile in the latter group, where synovial blood flow was significantly increased, suggesting increased angiogenesis and/or innervation.

RA as an inflammatory disease exhibits distinct stages (ie, acute/early RA vs chronic/established RA) characterised by different immunogenic signatures^32^ and symptoms.^33^ The leucocyte population in synovial lavage fluid following CFA-induced arthritis was assessed to investigate leucocyte infiltration into the synovium. We show that deletion of TRPC5 resulted in an unexpected, neutrophil-driven inflammation 14 days following induction of arthritis. The contribution of neutrophils in murine models of inflammatory arthritis is established,^34^ and in CFA-induced arthritis, neutrophil infiltration peaks earlier at 18–24 h following induction and is then substantially reduced by 14 days.^35^ In WT mice, we observed the typical mononuclear cell-driven inflammation 14 days post CFA-induced arthritis, which is similar to leucocyte populations found in patients with established arthritis.^36–38^ Mononuclear cellular infiltrate in the inflamed synovium did not differ between WT and TRPC5 KO mice; additionally, circulating leucocytes did not differ between WT and TRPC5 KO mice. Collectively, these results indicate that the protective role of TRPC5 is localised to the synovium, with neutrophils, in addition to mononuclear cells playing an important role in sustaining the inflammatory response.

We corroborated these results by measuring cytokine concentrations in synovial lavage fluid and in plasma samples and show that TRPC5 deletion or antagonism increased the local secretion of a number of critical pro-inflammatory cytokines (eg, TNFα, IL-1β) with an established role in joint disease. A positive correlation between disease activity and cytokine concentrations in clinical and animal models of arthritis has been previously established.^39^ ^40^ These cytokines are central to the pathogenesis of RA, leading a network of events including the recruitment and activation of inflammatory cells and propagation of joint destruction by inducing the secretion of MMPs.^17^ ^18^ In FLS cells, extracellular reduced thioredoxin was shown to directly stimulate TRPC5 and to suppress the secretion of MMPs,^10^ and in line with this, we observed an enhanced induction in the expression of MMPs in the inflamed synovium of TRPC5 KO mice, suggesting accelerated synthesis of these enzymes. Interestingly, we noted a significant increase in the immunoregulatory and anti-inflammatory cytokine, IL-10,^41^ ^42^ in TRPC5 KO and antagonist-treated WT mice, suggesting that IL-10 secretion was elevated to regulate the heightened inflammatory response.

In summary, our results provide evidence of a potential role for TRPC5 as a negative regulator of inflammation; in particular, the early response cytokine TNFα-neutrophil-innate immunity axis. We provide evidence that TRPC5 is protective in a murine model of arthritis, where genetic deletion/pharmacological blockade of TRPC5 sustained active joint inflammation, augmented hyperalgesia and synovitis. Taken together, our results suggest that activation of endogenous TRPC5 initiates a protective network against inflammatory insults and may facilitate novel therapeutic strategies for RA.
