# Supplementary material for: Transient receptor potential canonical 5 (TRPC5) protects against pain and vascular inflammation in arthritis and joint inflammation
Source: Ann Rheum Dis. 2016 May 10;76(1):252–60. doi: 10.1136/annrheumdis-2015-208886 (PMC5264234; doi:10.1136/annrheumdis-2015-208886)
Supplement: Supplementary data [file annrheumdis-2015-208886supp.pdf]

Online Supplementary Materials and Methods

Reagents

All drugs and reagents were from Sigma-Aldrich (Dorset, U.K.) unless otherwise stated.

Patients

Non-arthritic controls were selected from post mortem donors with no known history of arthritis or knee pain, as indicated by the next of kin and case notes review, and no knee osteophytes or Heberden's nodes on macroscopic examination. Inflammatory arthritis cases satisfied American College of Rheumatology criteria for rheumatoid arthritis (n=7),[1] or had an undifferentiated polyarthritis (n=1). OA cases satisfied ARA classification criteria,[2]. Histological synovitis scores were undertaken on haematoxylin and eosin stained tissue sections as previously described,[3].

Supplementary Table 1: Patient details.

	Non-arthritic controls	Inflammatory polyarthritis	Osteoarthritis
Number	8	8	8
Median age (range)	68 (61 – 87)	68 (61 – 88)	68 (61 – 90)
Sex, male/female (%)	37.5	25	50
Median synovium inflammation score (range)	0	2.5 (0.25 – 3)	2 (0 – 2.75)

Mice

Animals were housed under temperature (22±2°C)-controlled colony rooms maintained under filtered positive pressure ventilation on a 12-12 h light/dark cycle beginning at 07:00 GMT with free access to water and food. Male, age-matched 129S1/SvImJ wildtype (WT) and TRPC5 knockout (KO) were used at 8-12 weeks of age. TRPC5 WT and KO mice breeding pairs were bred from mice provided by Prof. D.E. Clapham (Howard Hughes Medical Institute, Boston, U.S.A) and were originally generated by recombineering,[4]. The genotype of each animal was established by PCR as previously described,[4]. All experiments were conducted under the guidelines of the United Kingdom Home Office Animals (Scientific Procedures) Act 1986 and were approved by the King's College London Animal Care and Ethics Committees.

Induction of joint inflammation

For induction of unilateral arthritis, mice were anaesthetised using isoflurane (2-3% carried in 2-3% O_2) and the hind-knee joint were injected with a single intra-articular (*i.art.*, 30G BD Micro-Fine insulin syringes) injection of Complete Freund's Adjuvant (CFA; 10 µg in 10 µl); the contralateral joint received 10 µl of saline (0.9% sodium chloride, pyrogen free; Baxter Healthcare, Thetford, U.K.). Joint inflammation was

allowed to progress over a period of 14 days. Behavioural measurements of hyperalgesia were obtained at baseline and stated time points. All experiments were conducted in a blinded manner and CFA/control injections were randomly assigned.

Behavioural measurements of hyperalgesia

Primary and secondary hyperalgesia, and weight-bearing assessments were carried out prior to induction of joint inflammation and on day 7 and 14 post-induction. Additionally, the diameter of knee joints was measured with callipers (Mitutoyo, Kanagawa, Japan) to assess joint oedema.

Primary hyperalgesia; pressure application meter (P.A.M.)

Primary mechanical hyperalgesia was measured at baseline, 7 days and 14 days post-induction of arthritis as previously described,[5, 6]. Briefly, mice underwent a habituation protocol prior to baseline measurements (results not recorded). A pressure transducer was connected to the acquisition software (PAM, Ugo Basile) to monitor the rate of force applied directly to each lateral side of the hind knee joint. Animals were loosely restrained by the operator and the pressure transducer was secured between the thumb and forefinger of the operator's hand. A steady force (30 g/f) was applied over 30 seconds (s) with a cut-off force of 500 g/f. Force application was stopped as soon as the animal displayed discomfort (e.g. flinching or movement of the hind knee). Measurements were obtained on two separate days to minimise stress and the mean responses were recorded.

Secondary hyperalgesia

For assessment of thermal hyperalgesia, hindpaw thermal nociceptive thresholds were determined before and at the stated times after administration of CFA, according to the Hargreaves method, as previously described,[7]. Briefly, the mice were housed in a behaviour box on a glass platform and allowed to acclimatize for 30 min before readings. The nociceptive threshold was measured with an automatic heat source (50W, 10V) directed onto the plantar surface of the paw and a timer linked to a light sensor. A cut-off time of 22 s was used to avoid tissue damage to the hindpaw. Measurements were obtained in triplicate, taken at least 5 min apart to minimise sensitisation, and the mean value was recorded as paw withdrawal latency in seconds (s).

Mechanical allodynia in the hindpaw were determined using a dynamic plantar aesthesiometer (Ugo Basile, Italy), an automated version of a von Frey hair device, as previously described,[8]. Mice were placed in observation boxes with a wire mesh floor and acclimatized for 30 min. Measurements were obtained with a straight metal filament (0.5 mm diameter), which exerts an increasing upward force (increasing by 1 g every 0.1 s) when touching the plantar surface of the hindpaw where withdrawal of the hindpaw stops the increasing force. Measurements were obtained in triplicate, and the mean value was used as the paw withdrawal threshold in grams (g).

Weight-bearing asymmetry

Weight-bearing was measured at baseline and at stated times thereafter. Asymmetry in weight bearing was measured as the difference in weight placed (g) between the ipsilateral hind limb and the contralateral control limb using an incapacitance meter (Linton Instruments, Norfolk, UK) as previously described,[9]. An average of five readings were obtained and the results expressed as (% weight placed on the ipsilateral limb/[ipsi + contra]).

Synovial blood flow measurement

In separate studies, blood flow in the synovial membrane was measured using a full-field laser perfusion imaging system (Moor Instruments, UK) 14 days following CFA-induced arthritis, as previously described,[10, 11]. Mice were anaesthetised with isoflurane (2% by air pump) on a homoeothermic heating blanket to regulate core body temperature and placed in a supine position. The skin overlying the knee joint was surgically excised and, in initial studies, the patellar tendon was also surgically removed to expose the underlying synovial membrane. Subsequently, synovial blood flow was measured with the patellar tendon intact, avoiding disruption to the joint. We found no difference in the flux value between exposed compared to intact studies. Blood flow was monitored in the ipsilateral and contralateral synovium over 5 min.

***Ex vivo* analysis**

Mice were anaesthetised with isoflurane (2-3% carried in 2-3% O_2) and blood samples were obtained via a cardiac puncture, using a heparinised syringe and needle (100 U/ml). Plasma was separated with centrifugation (400 x *g*, 20 min), snap frozen in liquid nitrogen and stored at –80°C until processing. Approximately 30 µl of synovial lavage fluid (SLF) was obtained by sequential flushing of the joint cavity as previously described,[12, 13].

Joint fixation and histological assessment

Mice were killed by cervical dislocation under isoflurane anaesthesia and the knee joint was dissected and trimmed, fixed for 1 week in 4% paraformaldehyfe and decalcified in 10% ethylenediaminetetraacetic acid in 10 mM Tris buffer (pH 6.95) for 2 weeks at room temperature,[14]. Joints were mounted in paraffin wax and sagittal sections (7µm) were cut and visualised using a 20x objective lens. Synovitis was assessed by blinded observer according to the methods described previously,[15].

Immunofluorescence staining

Double-immunofluorescence staining was carried out as previously described,[16]. Paraffin slides were deparaffinised by graded ethanol and xylene washes; heat mediated antigen retrieval was carried out by immersing the slides in 10mM citrate buffer (pH 6.0) at subboiling temperature for 10min.The slides were rinsed in PBS and and incubated with 10% (wt/vol) bovine serum albumin for 1h at room

temperature, followed by overnight incubation with primary antibodies at 4°C. Sections were incubated for 1h at room temperature with fluorescently tagged secondary antibodies, donkey anti-goat Alexa 488 (1:500, Invitrogen), or donkey anti-mouse Alexa 555 (1:500, Invitrogen). Negative controls omitted the primary antibody (see online figure S1). Slides were mounted with glass coverslips using Vectashield (Vector) medium containing 4,6-diamidino-2-phenylindole (DAPI) for nuclear identification. Fluorescent images were acquired using an Olympus BX51 microscope connected to an Olympus Colourview III camera. Alexa 488 was excited at 488 nm and emission collected at 505–550 nm, Alexa 555 excited at 555 nm and emission collected at >560.

Antibodies

Immunofluorescence detection of TRPC5 was performed using a mouse anti-TRPC5 (1:100, ab189262, Abcam). Sections were colabelled for the fibroblast-like synoviocyte (FLS) marker CD55, using goat anti-CD55 (1:50, sc-7067, Santa Cruz).

Cytokine concentration determination by multiplex

Plasma and SLF samples were thawed on wet ice, centrifuged (1000 x *g*, 5 min) and cytokine concentrations were determined using a commercial multiplex assay kit following manufacturer's protocol (V-PLEX Plus Proinflammatory Panel 1; Meso Scale Discovery, Rockville, MD, USA).

Cellular composition of synovial lavage fluid

In separate experiments, synovial lavage fluid (SLF) was collected as above for cytospin preparations to assess the cellular composition in the lavage fluid,[13] under isoflurane anaesthesia (2-3% carried in 2-3% O_2). Total cell counts were performed with an improved Neubauer haemocytometer under 40x objective after diluting an aliquot of SLF with haemolysis solution (Türk solution, 1:10; Fluka, UK). For differential cell counts, cytospin preparations were prepared with the remainder of samples (1:10 in PBS) centrifuged at 1000 rpm for 1 min using a Shandon Cytospin 2 (Shandon Southern Instruments, Sewickley, PA, USA) at room temperature. Cells were stained with Diff Quick (DADE Behring, Germany) and a total of 100 cells were counted by two blinded observers to determine the proportion of neutrophils and mononuclear cells using standard morphological criteria,[17]. Representative images (x100 magnification) were captured on a Leica DM 2000 LED microscope equipped with a Leica DFC294 camera, and acquisition software (Leica Application Suite Version 4.4).

Differentiation of peripheral blood (PB) leukocytes

In parallel with cytospin preparations of SLF, heparinized blood was obtained by cardiac puncture under isoflurane anaesthesia (2-3% carried in 2-3% O_2). Total leukocyte count was determined using an improved Neubauer haemocytometer under 40x objective following dilution of PB with haemolysis solution (Türk solution, 1:10; Fluka, UK). Cells were stained with Diff Quick (DADE Behring, Germany) and a

total of 100 cells were counted to determine the proportion of neutrophils and mononuclear cells using standard morphological criteria.

Quantitative Polymerase Chain Reaction (qPCR)

On termination of studies, the synovial membrane was dissected and immersed in *RNALater* RNA Stabilisation Reagent (Qiagen, UK) and stored at –20°C. RNA was extracted using the RNEasy Microarray kit according to manufacturer's instructions (Qiagen). First-strand complementary DNA (cDNA) was synthesised from 200ng of total RNA using the High-Capacity RNA-cDNA kit supplemented with RNAse inhibitors according to manufacturer's instructions (Applied Biosystems, UK),[18].

Human synovium was snap frozen and RNA was extracted using the RNEasy Microarray kit according to manufacturer's instructions (Qiagen). Total RNA (1000ng) was reverse transcribed into complementary DNA (cDNA) as described above. Primers were designed in-house using Primer Blast software (http://www.ncbi.nlm.nih.gov/tools/primer-blast/), and synthesised by Sigma; details of primers and PCR product sizes are listed in supplementary table 2 and supplementary table 3. Quantitative PCR (qPCR) was performed with the SensiMix SYBR No-ROX Kit (Bioline, London, United Kingdom) with Hot-Start Taq polymerase on a Corbett Rotorgene 6000 (Qiagen, UK). Samples were heated to 95°C for 10 min (initial denaturation), followed by 45 cycles of 10 s at 95°C, 15 s at 57°C, and 5 s at 72°C; melt at 68–90°C with fluorescence detection after each cycle. Samples were subjected to melting curve analysis to confirm product specificity. Expression of each gene was analysed as copy/µl derived from a standard curve using the Rotorgene 6000 software. Results were normalised to geometric mean of hypoxanthine guanine phosphoribosyl transferase (HPRT), β-actin and phospholipase A_2 (PLA_2) using GeNorm version 3.4; see Smillie *et al.*, 2014 and Fernandes *et al.*, 2016 for full details [19, 20]

Supplementary Table 2 Mouse primer sequences used in qPCR studies.

Gene symbol	***Primer Sequence***	***Accession number***	***Product length (base pairs)***
Actb	F: CACAGCTTCTTTGCAGCTCCTT<br>R: TCAGGATACCTCTCTTGCTCT	NM_007393.5	250
Ccl 2	F: GAAGCTGTAGTTTTTGTCACCA<br>R: TTCCTTCTTGGGGTCAGCAC	NM_011333.3	60
Cd3e	F: CGTCCGCCATCTTGGTAGAGAGAGCAT<br>R: CTACTGCTGTCAGGTCCACCTCCAC	NM_007648.4	373
Hprt	F: TCCTCCTCAGACCGCTTT T<br>R: CCTGGTTCATCATCGCTAATC	NM_013556.2	90
Mmp2	F: GAC CAGGTTATCAGGGATGGCATTC<br>R: AAGTTCTGGAGATACAATGAAGTG	NM_006530751.1	96
Mmp3	F: CCCACATCACCTACAGGATTGT<br>R: GACTGTTCCAGGCCCATCAA	NM_010809.1	206
Mmp13	F: ACAAGCAGTTCCAAAGGCTACA<br>R: GCTGGGTCACACTTCTCTGG	NM_008607.2	239
Nfkb1	F: AGGATTTGCTGAGGGTTGGG<br>R: TGGCATTTAGACCTTCCCCAT	NM_008689.2	168
Pla2g12a	F: TGGATATAAACCATCTCCACCA<br>R: GGGAAGGGATACCTATGTTCAGA	NM_023196.4	77
Timp2	F: TAATTGCAGGAAAGGCAGAAGGA<br>R: GGGAGGAGATGTAGCAAGGG	NM_011594.3	174
Timp3	F: ATCGTGATCCGGGCCAAAG<br>R: GAAGCCTCGGTACATCTTCATCT	NM_011595.2	102
Timp4	F: GCTCTAGTGATACGGGCCAA<br>R: TTGGCCTTCTCGAACCCTTT	NM_080639.3	131
Tnf	F: CTGTAGCCCACGTCGTAGCAAA<br>R: GACGGCAGAGAGGAGGTTGA	NM_013693.3	254
Trpc1	F: TTGCGTAGATGTGCTTGGGA<br>R: TTGCGTAGATGTGCTTGGGA	NM_011643.2	101
Trpc4	F: TACGGAAACCCCATCGGAAC<br>R: AGTCCATCATCATCTCCGCAA	NM_001253683.1	128
Trpc5	F: GGAGATAAAGGAAATGTGGGATGGT<br>R: AATAGTTGCCAGGTAGAGGGAGT	NM_009428.2	100
Trpc6	F: CCAGCTTCCGGGGTAATGAA<br>R: TAGCATCTTCCGCACCACTG	NM_013838.2	191

F: Forward, R: Reverse. Actb; beta actin, CCL2; chemokine (C-C motif) ligand 2, CD3; CD3 antigen, epsilon polypeptide, , HPRT; hypoxanthine guanine phosphoribosyl transferase, MMP2; matrix metallopeptidase 2, MMP3; matrix metallopeptidase 3, MMP13; matrix metallopeptidase 13, NF-κB; nuclear factor of kappa light B1, p105, PLA_2; phospholipase A2, group XIIA, TAC1; tachykinin 1, TIMP2; tissue inhibitor of metalloproteinase 2, TIMP3; tissue inhibitor of metalloproteinase 3, TIMP4; tissue inhibitor of metalloproteinase 4, TNF; tumour necrosis factor, TRPC1; transient receptor potential cation channel, subfamily C, member 1, TRPC4; transient receptor potential cation channel, subfamily C, member 4, TRPC5; transient receptor potential cation channel, subfamily C, member 5, TRPC6; transient receptor potential cation channel, subfamily C, member 6.

Supplementary Table 3 Human primer sequences used in qPCR studies.

Gene symbol	*Primer Sequence*	*Accession number*	*Product length (base pairs)*
ACTB	F: AGGCATCCTCACCCTGAAGT<br>R: GTTGAAGGTCTCAAACATGATCTGG	NM_001101.3	199
B2M	F: AGATGAGTATGCCTGCCGTG<br>R: TCATCCAATCCAAATGCGGC	NM_004048.2	120
RPL13A	F: AGAAGCCCTTTGAGGAGCA<br>R: CGATTACGGGTCTATATTCCAGA	NM_001270491.1	112
TNFRSF1A	F: AGTTGTGCCTACCCCAGATTG<br>R: GCTCCCCCTCTTTTTCAGGTG	NM_001065.3	198
TRPC5	F: GTTCTAGGTTTCATTTGGGGTGAGA<br>R: ACATTTCCCATTCCTCCCTTGG	NM_012471.2	184
VCAM1	F: GGATAATGTTTGCAGCTTCTCAAG<br>R: TTCGTCACCTTCCCATTCAGT	NM_080682.2	171

F: Forward, R: Reverse. ACTB; beta actin, B2M; beta-2-microglobulin, RPL13A; ribosomal protein L13a, TNFRSF1; tumor necrosis factor receptor superfamily member 1A, TRPC5; transient receptor potential cation channel, subfamily C, member 5, VCAM1; vascular cell adhesion molecule 1 (VCAM1).

Supplementary Table 4.

**Target**	**TRPC5 WT**		**TRPC5 KO**	
	**Saline**	**CFA**	**Saline**	**CFA**
TRPC1	20.2 ± 0.6	30.6 ± 3.8	34.8 ± 10.2	19.1 ± 3.3
TRPC3	8.8 ±2.5	13.9 ±3.8	9.7 ± 0.1	10.5 ± 2.3
TRPC4	ND	ND	ND	ND
TRPC6	38.6 ± 7.6	56.5 ± 11.2	49.7 ± 12.2	33.3 ± 6.7

Investigating TRPC channel expression in the synovial membrane following CFA-induced arthritis.

Real-time quantitative PCR analysis of the expression of transient receptor potential canonical 1 (TRPC1), transient receptor potential canonical 4 (TRPC4) and transient receptor potential canonical 6 (TRPC6) in the contralateral and ipsilateral of TRPC5 WT (n=8) and KO (n=7) mice, normalized to HPRT, β-actin and PLA_2, 14 days post-induction of arthritis. $p>0.05$ as determined by 2-way ANOVA + Bonferroni *post hoc* test; values are mean ± S.E.M. ND = not detected.

Antagonist treatment groups

TRPC5 WT and TRPC5 KO mice were treated with the TRPC4/5 antagonist ML204 (2mg/kg; Tocris, Bristol, U.K.) or vehicle (2% DMSO in saline, *i.p.*) 1 h prior to induction of joint inflammation. Daily treatments were continued for 2 weeks (see online figure S3A); joint inflammation was assessed as described above. This dose was chosen based on preliminary testing and found to be well-tolerated with no adverse effect observed on the animals.

Statistical Analysis

Results are expressed as mean ± standard error of the mean (S.E.M.) and analysed by two-way ANOVA and Bonferroni *post hoc* test using Graph Pad Prism 5.0 (San Diego California, USA, unless stated. Significance was accepted as $P<0.05$.

References

1. Arnett FC, Edworthy SM, Bloch DA*, et al.* The American Rheumatism Association 1987 revised criteria for the classification of rheumatoid arthritis. *Arthritis Rheum* 1988; 31(3):315-324.

2. Altman R, Asch E, Bloch D*, et al.* Development of criteria for the classification and reporting of osteoarthritis. Classification of osteoarthritis of the knee. Diagnostic and Therapeutic Criteria Committee of the American Rheumatism Association. *Arthritis Rheum* 1986; 29(8):1039-1049.

3. Haywood L, McWilliams DF, Pearson CI*, et al.* Inflammation and angiogenesis in osteoarthritis. *Arthritis Rheum* 2003; 48(8):2173-2177.

4. Riccio A, Li Y, Moon J*, et al.* Essential role for TRPC5 in amygdala function and fear-related behavior. *Cell* 2009; 137(4):761-772.

5. Amorim D, David-Pereira A, Pertovaara A*, et al.* Amitriptyline reverses hyperalgesia and improves associated mood-like disorders in a model of experimental monoarthritis. *Behav Brain Res* 15; 265:12-21.

6. Barton NJ, Strickland IT, Bond SM*, et al.* Pressure application measurement (PAM): a novel behavioural technique for measuring hypersensitivity in a rat model of joint pain. *J Neurosci Methods* 2007; 163(1):67-75.

7. Russell FA, Fernandes ES, Courade JP*, et al.* Tumour necrosis factor alpha mediates transient receptor potential vanilloid 1-dependent bilateral thermal hyperalgesia with distinct peripheral roles of interleukin-1beta, protein kinase C and cyclooxygenase-2 signalling. *Pain* 2009; 142(3):264-274.

8. Fernandes ES, Russell FA, Spina D*, et al.* A distinct role for transient receptor potential ankyrin 1, in addition to transient receptor potential vanilloid 1, in tumor necrosis factor alpha-induced inflammatory hyperalgesia and Freund's complete adjuvant-induced monarthritis. *Arthritis Rheum* 2011; 63(3):819-829.

9. Ashraf S, Mapp PI, Burston J*, et al.* Augmented pain behavioural responses to intra-articular injection of nerve growth factor in two animal models of osteoarthritis. *Annals of the rheumatic diseases* 2014; 73(9):1710-1718.

10. Aubdool AA, Graepel R, Kodji X*, et al.* TRPA1 is essential for the vascular response to environmental cold exposure. *Nat Commun* 2014; 5:5732.

11. Keeble JE, Brain SD. Capsaicin-induced vasoconstriction in the mouse knee joint: a study using TRPV1 knockout mice. *Neurosci Lett* 2006; 401(1-2):55-58.

12. Keeble J, Russell F, Curtis B*, et al.* Involvement of transient receptor potential vanilloid 1 in the vascular and hyperalgesic components of joint inflammation. *Arthritis Rheum* 2005; 52(10):3248-3256.

13. Baddack U, Hartmann S, Bang H*, et al.* A chronic model of arthritis supported by a strain-specific periarticular lymph node in BALB/c mice. *Nat Commun* 2013; 4:1644.

14. Ashraf S, Mapp PI, Walsh DA. Contributions of angiogenesis to inflammation, joint damage, and pain in a rat model of osteoarthritis. *Arthritis Rheum* 2011; 63(9):2700-2710.

15. Krenn V, Morawietz L, Burmester GR*, et al.* Synovitis score: discrimination between chronic low-grade and high-grade synovitis. *Histopathology* 2006; 49(4):358-364.

16. Alfieri A, Srivastava S, Siow RC*, et al.* Sulforaphane preconditioning of the Nrf2/HO-1 defense pathway protects the cerebral vasculature against blood-brain barrier disruption and neurological deficits in stroke. *Free Radic Biol Med* 2013; 65:1012-1022.

17. Baldissera-Jr L, Monteiro PF, de Mello GC*, et al.* Platelet adhesion and intracellular calcium levels in antigen-challenged rats. *Pulm Pharmacol Ther* 2010; 23(4):327-333.

18. Alawi KM, Aubdool AA, Liang L*, et al.* The sympathetic nervous system is controlled by transient receptor potential vanilloid 1 in the regulation of body temperature. *FASEB J* 2015 Jul 1.

19. Fernandes ES, Russell FA, Alawi KM*, et al.* Environmental cold exposure increases blood flow and affects pain sensitivity in the knee joints of CFA-induced arthritic mice in a TRPA1-dependent manner. *Arthritis Res Ther* 2016; 18(1):7.

20. Smillie SJ, King R, Kodji X*, et al.* An ongoing role of alpha-calcitonin gene-related peptide as part of a protective network against hypertension, vascular hypertrophy, and oxidative stress. *Hypertension* 2014; 63(5):1056-1062.

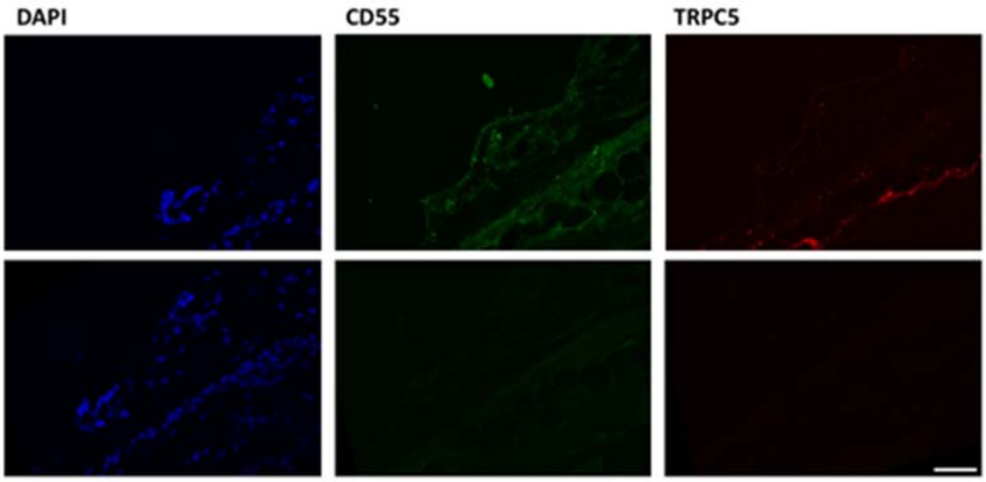

Figure S1.
Negative control
Top panel demonstrates positive staining for CD55 (middle panel) and TRPC5 (right panel) in mouse synovium. Bottom panel demonstrates negative control fluorescence secondary antibody staining for CD55 (donkey anti-goat Alexa Fluor 488, middle panel) and TRPC5 (donkey anti-mouse Alexa Fluor 555, right panel). DAPI (blue) was used to identify nuclei; scale bar represent 50µm.

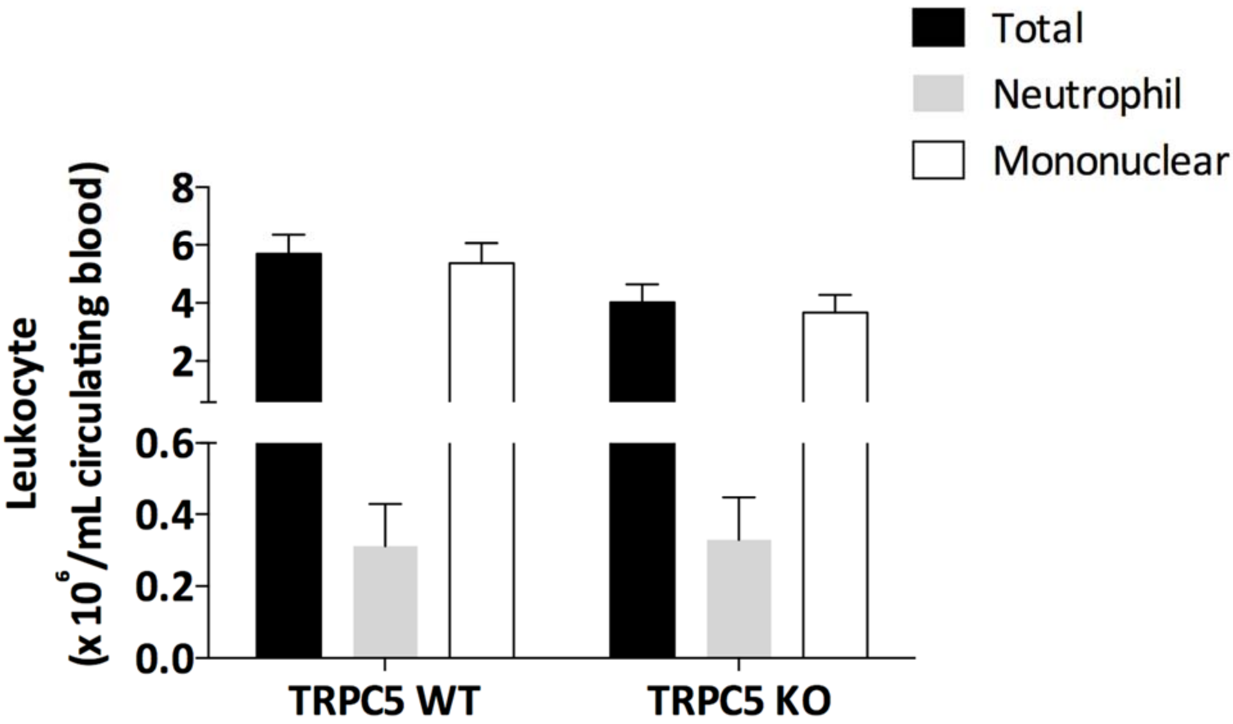

Figure S2.

Peripheral blood leukocytes following CFA-induced arthritis.

PB was collected at day 14 following CFA-induced arthritis in WT and TRPC5 KO mice (n=7) and leukocytes (total, neutrophils and mononuclear cells) were determined by differential counting of blood smears. $p>0.05$ as determined by 2-way ANOVA + Bonferroni *post hoc* test;values are mean ± S.E.M.

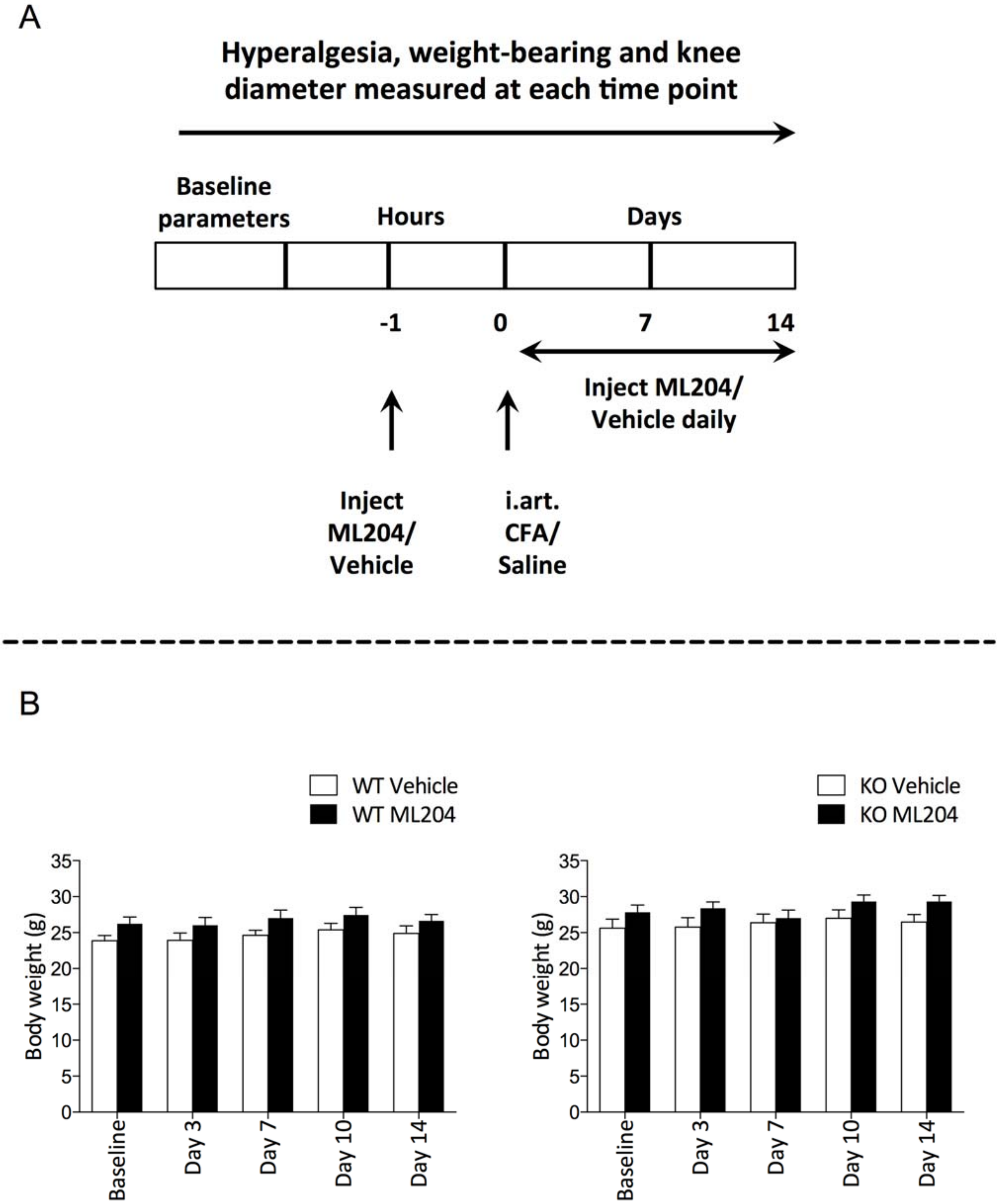

Figure S3.

Schematic representation of chronic pharmacological treatment studies.

(A) Time-line illustrating experimental design of chronic pharmacological studies in TRPC5 WT and KO mice. (B) Body weight of vehicle treated (n=5) and ML204 treated mice (n=6) over 14 days. $p>0.05$ as determined by 2-way ANOVA + Bonferroni *post hoc* test; values are mean ± S.E.M.

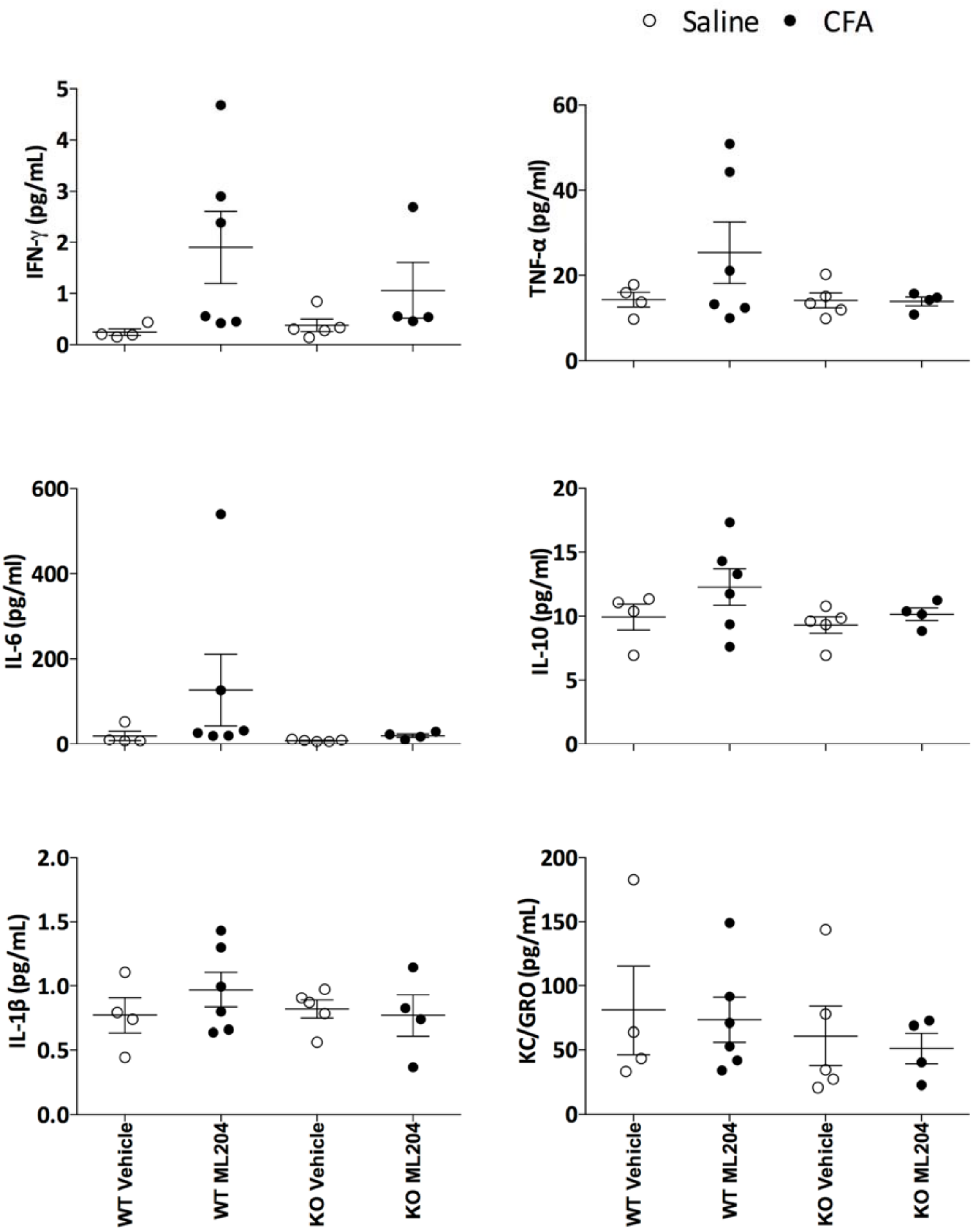

Figure S4.

Circulating cytokines following CFA-induced arthritis.

Cytokine concentrations in plasma 14 days following CFA-induced arthritis in in vehicle (2% DMSO in saline, *i.p.*) treated WT and TRPC5 KO mice (n=5), ML204-treated WT and TRPC5 KO mice (2mg/kg, *i.p.*, daily n=6). Interferon-gamma (IFN-γ), tumour necrosis factor-α (TNFα), interleukin 6 (IL-6), interleukin 10 (IL-10) and inerleukin 1β (IL-1β), keratinocyte chemoattractant (KC). $p>0.05$ as determined by 2-way ANOVA + Bonferroni *post hoc* test; values are mean ± S.E.M.

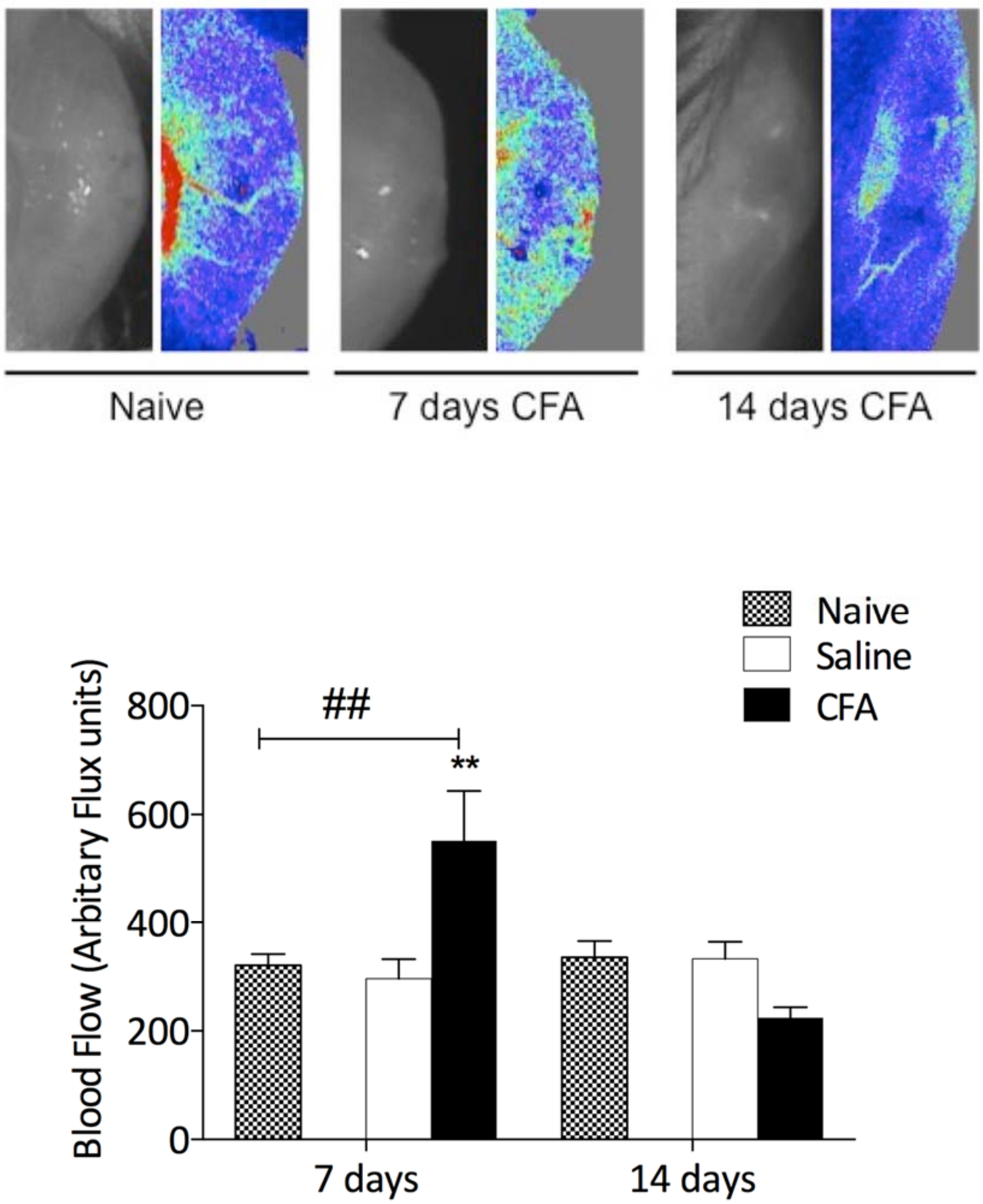

Figure S5.
Synovial blood flow following CFA-induced arthritis.
Characterisation of vascular effects of CFA-induced arthritis assessed by full-field laser perfusion imaging (FLPI) on day 7 (n=4) and day 14 (n=4). Naïve, untreated mice were assessed on each occasion (n=4) as a control. **p<0.01 vs control; ##p<0.01 vs naïve by 2-way ANOVA + Bonferroni *post hoc* test; values are mean ± S.E.M.
